# Supplementary material for: 3D Super-Resolution Nuclear Q-FISH Imaging Reveals Cell-Cycle-Related Telomere Changes
Source: Int J Mol Sci. 2024 Mar 10;25(6):3183. doi: 10.3390/ijms25063183 (PMC10970233; doi:10.3390/ijms25063183)
Supplement: Supplementary file 1 [file ijms-25-03183-s001.zip › ijms-2870639-supplementary.pdf]

Supplementary Figures and Protocols

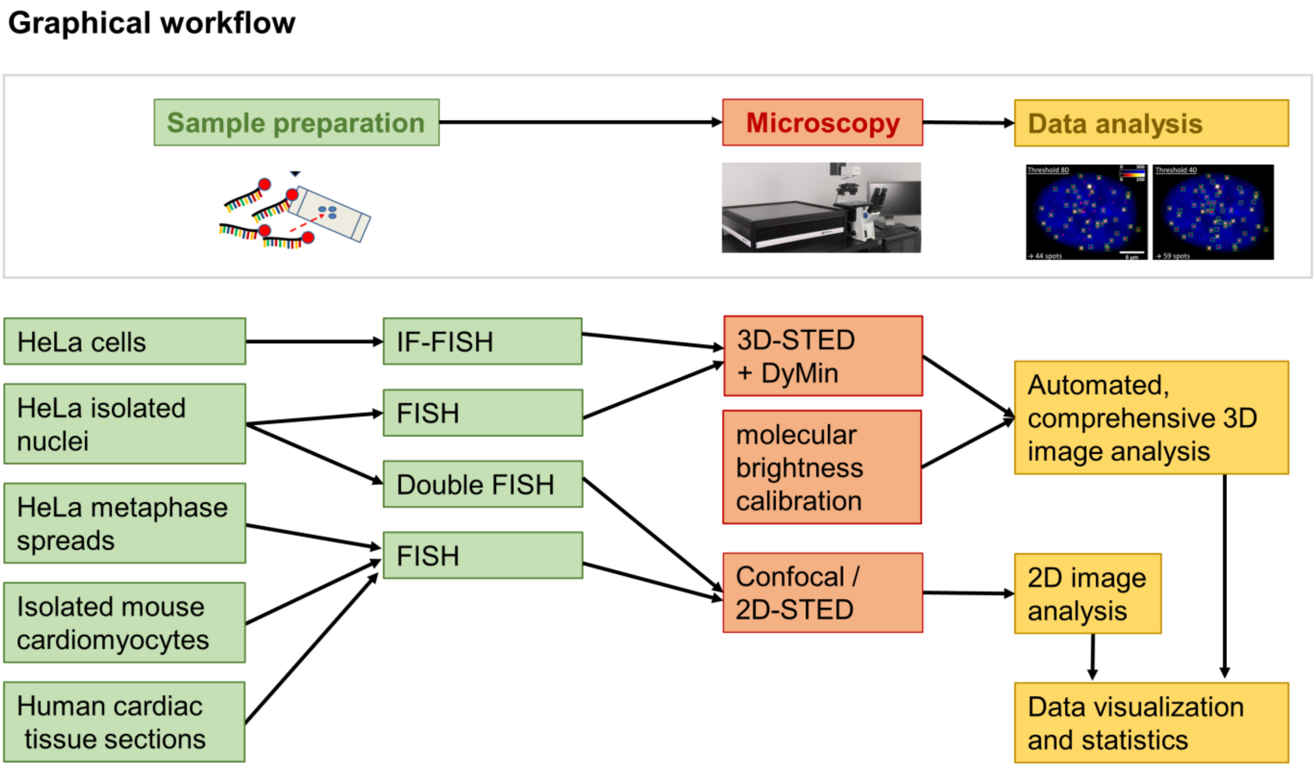

Figure S1. Graphical workflow of the study.

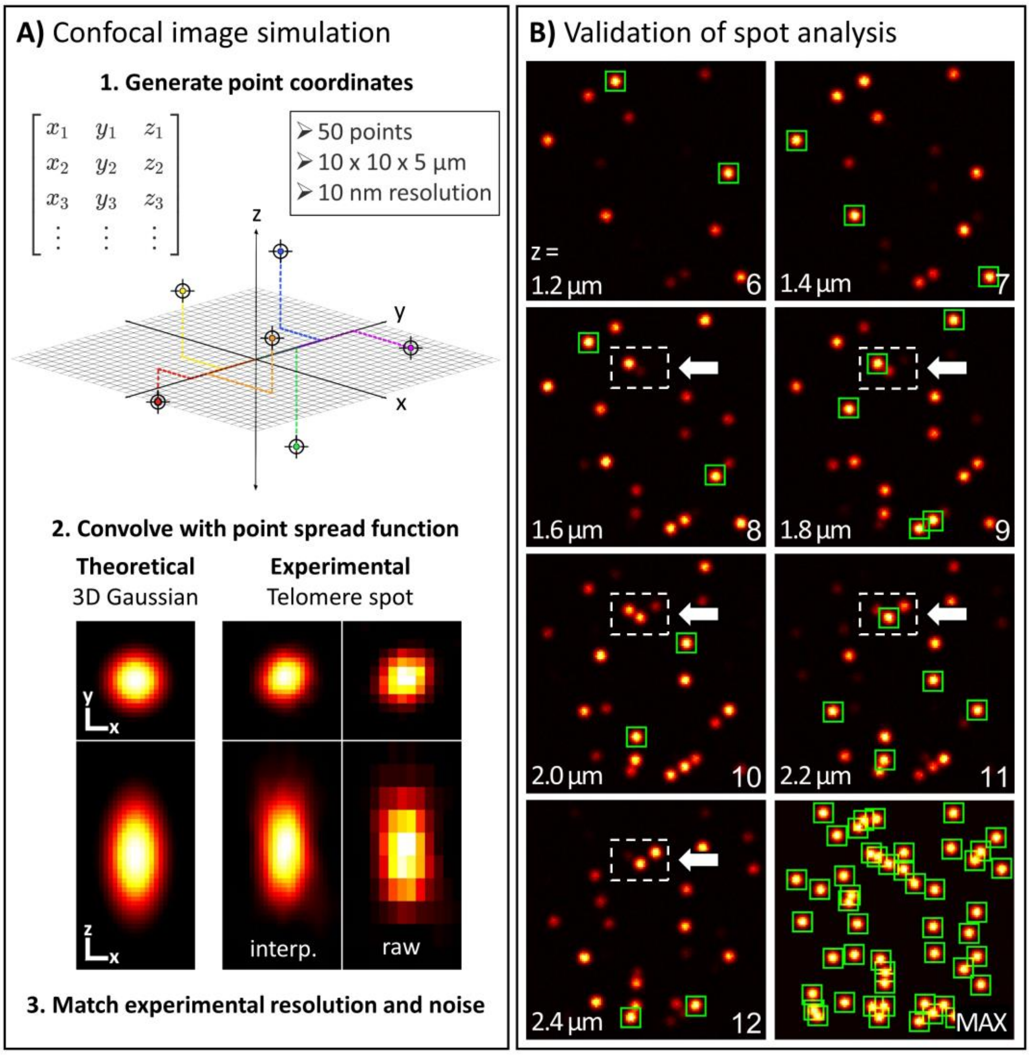

Figure S2. Simulation of synthetic microscopy data for validation of image analysis.

A) Points (n = 50) were randomly placed in 3D space and convolved with a theoretical Gaussian PSF with dimensions instructed by measured telomere spots inside of nuclei. For comparison, a simulated spot and a measured telomere spot are shown (scale bar = 200 nm, pixel size 40 nm, bicubic interpolation). The generated images were then mapped to the experimental image resolution and noise was added. (N = 10 images). B) An example of spot detections (green squares) in subsequent slices (7 of 25) of simulated 3D telomere data resulting from our analysis routine. Even partly overlapping spots are resolvable, as can be seen in the marked region. The maximum intensity Z projection (MAX) shows that in this case, all 50 spots were correctly detected and localized.

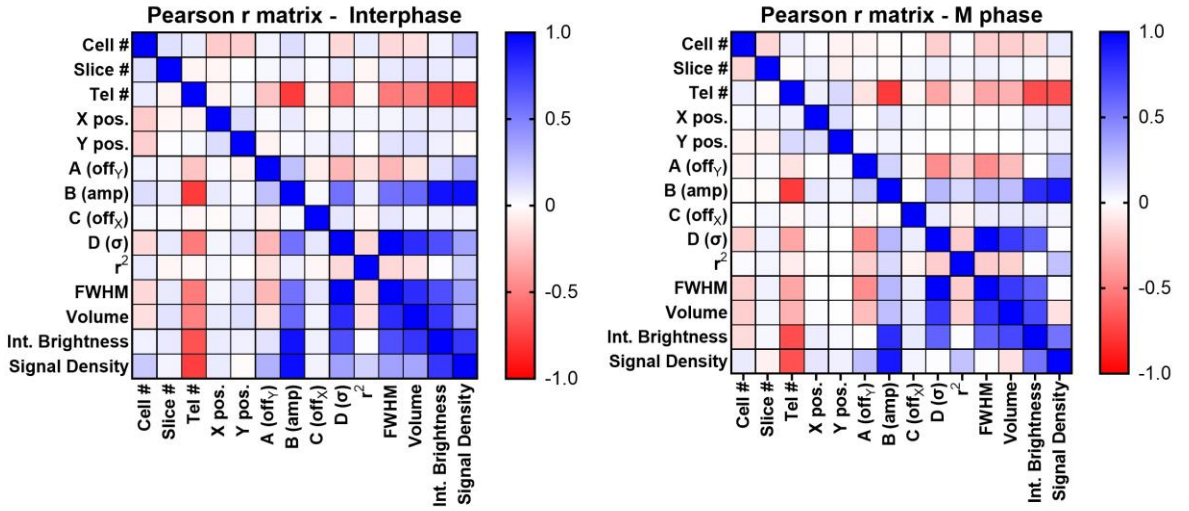

Figure S3. Telomere parameter correlation matrix confirms absence of clustered data and telomere subpopulations.

Data from HeLa interphase cells (773 telomeres) was used to generate a correlation matrix for telomere parameters. Most unlinked variables are uncorrelated confirming unbiased telomere detection and analysis. There is no evidence for a population subset or spatial telomere clustering. The last four variables are permutations of size and brightness and thus show spurious correlations. Given the high coverage of telomeres this argues against subpopulations in DNA packing ratio and clustering.

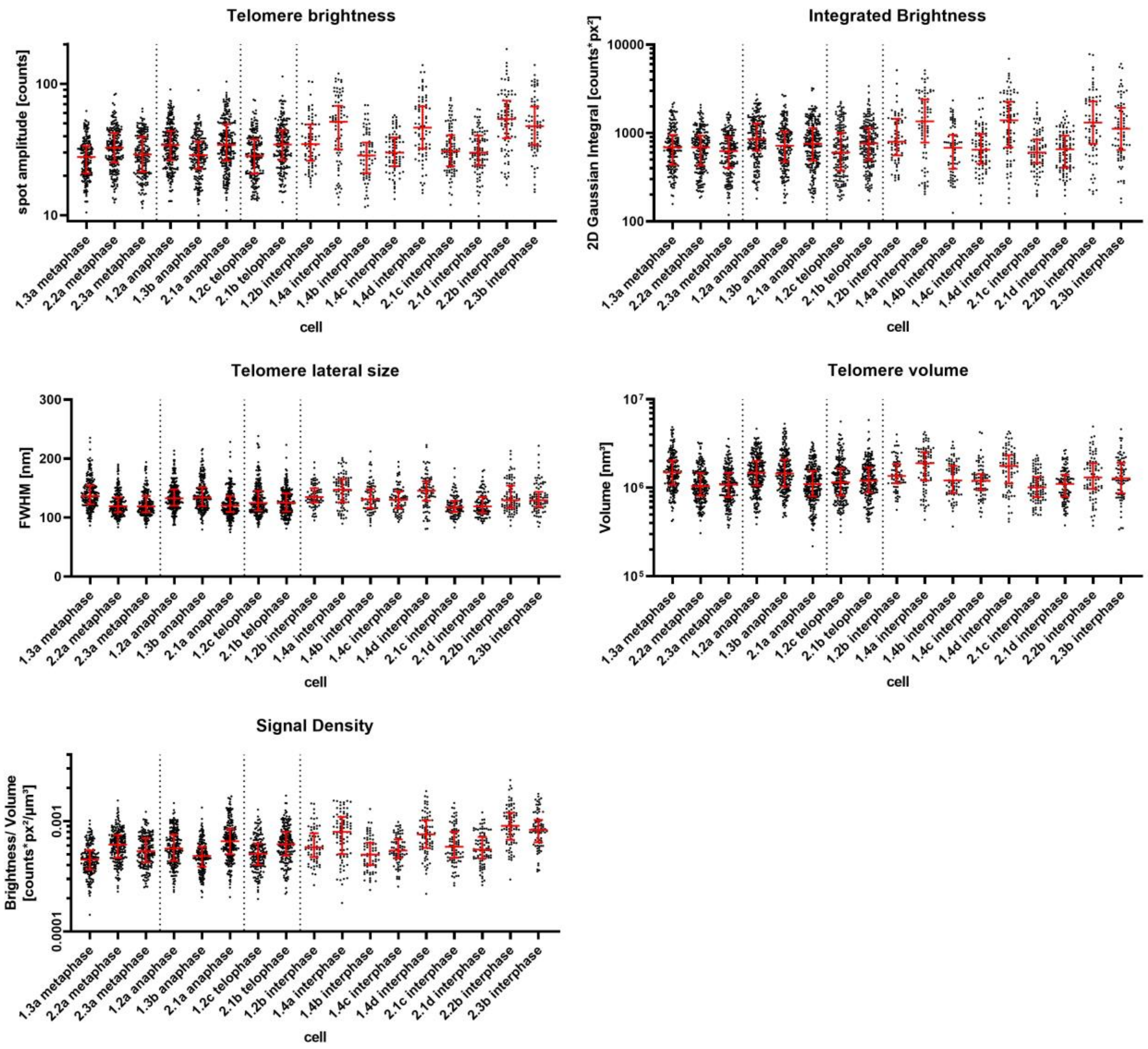

Figure S4. **Raw data of telomere spot analysis by cell.**

Each point represents a single telomere. Red lines indicate median and IQR. Cell numbering indicates slide and region number, while letters identify single cells. Dashed lines separate metaphase, anaphase, telophase and interphase cells.

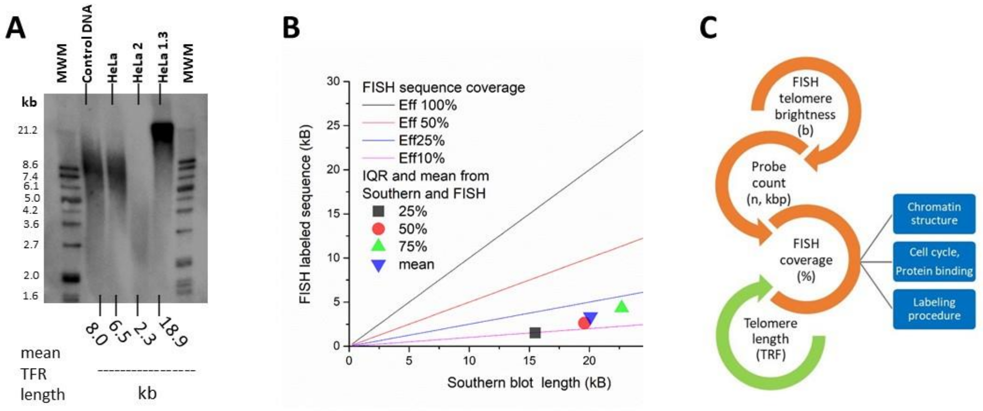

Figure S5: Comparison of TRF data with telomere FISH-STED nanoscopy sheds light on FISH labeling and detection efficiencies.

A) TFR Southern blots on different Hela lines confirmed published mean telomere lengths. Mean telomere lengths (bottom) are indicated by arrows. B) A correlation plot shows the distribution of telomere lengths represented in Southern blots compared to the distribution of chromosome DNA sequence lengths covered by TelC probes. TelC probe occupied sequence lengths were extrapolated from TelC-probe molecule counts per telomere in Hela 1.3. Telomere lengths extrapolated from PNA-probe counts represent only a small fraction of telomere sequence detected by Southern blotting, ranging between 10 to 20% of coverage. Straight lines indicate the correlation of telomere lengths from Southern blots with FISH labeled telomere sequence for different levels of binding efficiency, expressed as sequence coverage. C) The workflow rationale is summarized as a cartoon. Possible factors affecting FISH coverage include chromatin structure, protein binding and labeling procedure. A classic method used for measuring telomere length is Terminal Restriction Fragment (TRF) length analysis[34] - the gold standard for telomere length measurement that yields accurate and well-comparable results, as they are expressed in absolute units (kbp).

Supplementary Protocol 1. Steps applied in the study

I. Sample preparation

A) Intact cultured cells (HeLa)
B) Isolated nuclei
C) Metaphase spreads
D) Freshly isolated mouse cardiomyocytes
E) Paraffin-embedded cardiac sections

II. Sample pretreatment

1. Rehydration, fixation
2. RNAse and pepsin pretreatment,
3. Deparaffinization and dehydration (for paraffin-embedded specimens)

III. FISH procedure

1. DNA denaturation and subsequent hybridization with PNA-probe
2. Washing and mounting; DAPI staining

IV. **IF-FISH procedure** (alternatively)

1. Fixation, permeabilization and incubation with primary and secondary antibodies
2. Fixation, dehydration and FISH procedure
3. Mounting, DAPI staining

V. Microscopy

1. Microscopic cell selection: Using a custom-built optical setup based on Abberior RESOLFT QUAD P microscope kit
2. Data acquisition using 2D or 3D-STED and Dynamic Intensity Minimum STED mode (DyMIN)
3. Molecular brightness calibration using commercial fluorescent bead slides

VI. Automated Image analysis

1. Preprocessing and spot detection using ImageJ plugin software FindFoci
2. Automated line profile spot analysis using ImageJ macro

VII. Data handling and statistics
